# Reduction in sugar intake after the introduction of minimum unit pricing for alcohol in Scotland: a difference-in-differences analysis

**DOI:** 10.1016/j.ajcnut.2025.101128

**Published:** 2025-11-27

**Authors:** Attakrit Leckcivilize, Stephen Whybrow, Ni Gao, Daniel Kopasker, Paul McNamee, Anne Ludbrook

**Affiliations:** 1 Health Economics Research Unit, University of Aberdeen, Aberdeen, United Kingdom; 2 Health Systems Collaborative, Nuffield Department of Clinical Medicine, University of Oxford, Oxford, United Kingdom; 3 Rowett Institute, University of Aberdeen, Aberdeen, United Kingdom; 4 Social & Public Health Science Unit, University of Glasgow, Glasgow, United Kingdom

**Keywords:** minimum unit pricing, alcohol, diet quality, nutrients, sugar, difference-in-differences

## Abstract

**Background:**

In 2018, Scotland introduced a minimum unit pricing (MUP) policy to remove very-low-cost alcoholic drinks from the market in an effort to reduce the adverse impacts of excessive alcohol consumption. Any increased spending on alcohol may be associated with reduced food and lower diet quality.

**Objectives:**

This study aimed to estimate the relationship between MUP and dietary energy, nutrients, and diet quality.

**Methods:**

Difference-in-differences analyses were conducted on household-level purchase data, collected by Kantar Worldpanel (KWP) over 53 wk before and 54 wk after the implementation of MUP, from 1987 households in Scotland and 6064 households in the north of England. The Poisson pseudomaximum likelihood regression model with household fixed effects was used, with estimates adjusted for age of main shopper, household composition, duration of KWP participation, total spending on nonfood items, and month of the year. Primary outcomes were dietary energy, energy density, Diet Quality Index, and foods and nutrients relevant to the Scottish dietary goals after adjustment to per adult-equivalent values. Secondary outcomes explored the differential effects of MUP by area-level deprivation and levels of alcohol purchase.

**Results:**

The introduction of MUP in Scotland was associated with a 1.6% [95% confidence interval (CI): 0.02%, 3.16%] reduction in the purchase of sugar from food and beverages or 8 g per adult equivalent per week. This reduction was partly a result of a 16.6% (95% CI: 7.15%, 25.96%) reduction in sugar from alcoholic drinks purchased. No other significant associations were found. Households from more deprived areas, and households with greater alcohol purchases, had greater levels of sugar reduction from alcohol.

**Conclusions:**

MUP in Scotland is associated with small, but beneficial, statistically significant reductions in the purchase of sugar. There is no significant change in overall diet quality.

## Introduction

Excessive alcohol consumption is a major source of health problems [1], with 5.1% of the global burden of disease attributed to alcohol [2]. Scotland has a higher level of alcohol-related harms than other parts of the United Kingdom. For example, Scotland had higher alcohol-specific death rates (22.6 deaths/100,000 persons) than the United Kingdom mean of 16.6 per 100,000 in 2022 [3]. In past decades, a range of policies, including banning multibuy price discounts and some promotional offers, has been introduced to tackle Scotland’s alcohol problems.

On 1 May, 2018, minimum unit pricing (MUP) for alcohol was implemented in Scotland, the first country to set a strength-based floor price for alcohol [4]. The legislation aimed to reduce alcohol consumption and adverse social and health impacts from alcohol-related harms by imposing a minimum price of 50 pence per unit (8 grams) of pure alcohol [5]. The policy was extended by the Scottish Parliament [6] following a report on the impact of MUP covering alcohol consumption and other health and social costs and benefits, including the potential impact on household food expenditure and diet quality [7].

Evidence suggests increasing alcohol intake is associated with higher energy intake [[8], [9], [10], [11], [12], [13]] and lower diet quality score [9,14]. Frequent heavy drinking is associated with lower carbohydrate intakes [15]. Changes in the relative prices between some previously cheap alcoholic drinks and other food and drink purchases could lead to reductions in diet quality if increased spending on alcoholic drinks displaces healthier foods; MUP mainly affects the price of alcohol sold in supermarkets, convenience stores, and other premises licensed to sell alcohol for consumption off the premises (off sales). Alcohol sold for consumption in bars and restaurants (on sales) had been the subject of previous policy interventions and would mostly be above the MUP threshold. Off sales alcohol accounts for most alcohol purchased in the United Kingdom (72.5% by volume in Scotland in 2017) [16]. From an economic perspective, given a constrained budget, and with off sales alcohol frequently purchased in the same basket of goods as food, an increase in expenditure on alcohol may affect expenditure on food through the complementarity between these 2 products [17].

Previous research identified a small, statistically significant reduction in food expenditure caused by MUP and a nonsignificant reduction in total volume of food purchased [18]. This study, for the first time, examines the association between MUP for alcohol and changes in the nutritional components of food purchased for consumption at home by households (primary outcomes). The effects of deprivation and level of alcohol purchasing are also examined (secondary outcomes). Given that MUP is implemented in Scotland but not in England, we compare the nutritional components of food purchases and diet quality 12 mo before and after the implementation of MUP between Scotland (treatment group) and north of England (control group). Our findings contribute to the wider evaluation of MUP and add to the literature on the nutritional relationship between food and alcohol.

## Method

### Data

We used large-scale household consumer panel data collected by the United Kingdom Kantar Worldpanel (KWP) and focused on energy intake, dietary energy density, intakes for fruit and vegetables, fiber, oil-rich fish, total carbohydrate, red and processed meat, salt, percentage energy intake from fat, saturated fat, and sugars, and Diet Quality Index (DQI). Data are anonymized, and informed consent was received by the original data collectors. All participants have opted into the panel and receive a small reward for submitting weekly data. The panel is designed by KWP to provide a representative sample of the population of Scotland and England. These data include weekly purchases of all food and drink brought into the home by panel members; items such as restaurant meals, takeaway food and on-premises alcoholic drinks are not included. Households used a handheld scanner device provided by KWP to scan a product’s barcode. Non-bar-coded items (e.g., fruit and vegetables) were also recorded. The price of each purchased product was collected using households’ till receipts. For each recorded product purchased, the data include the description of the product, type of product (e.g., bread), quantity (weight or volume), and amount of money spent. For purchases of alcoholic drinks, the number of units of alcohol for each purchase was calculated from volume purchased and alcohol content [alcohol by volume (ABV), where 1 unit of alcohol equals 8 g pure alcohol]. The ABV was detailed in the product description for most products, but where absent, a standard ABV value was used according to the type of alcoholic product (e.g., 40% for whiskey).

### Nutrient intakes and DQI score (outcome data)

The primary outcomes were energy, fruit and vegetables, oil-rich fish, total carbohydrate, red and processed meat, sugar, sugar excluding alcohol, sugar including alcohol only, fat, saturated fat, salt, fiber, energy density (kcal/100 g), and DQI. Nutritional information was collected by KWP from product labels, food composition tables, or product group averages. Composite foods were disaggregated to estimate the proportion of foods relevant to the Revised Dietary Goals for Scotland, such as fruit, vegetables and oil-rich fish. Detailed definitions of each nutrient and disaggregation were described in Whybrow et al. [19].

Energy density (kcal/100 g) of the food purchased was calculated from the contribution of all food and milks, but excluded all drinks (tea, coffee, water, fruit juices, squashes, sugar-containing drinks, artificially sweetened drinks, and alcohol). This is the same method as used in setting the Revised Dietary Goals for Scotland [20] and others [21,22].

A DQI was calculated for each household from each week’s food and drink purchases to allow comparison against the Revised Dietary Goals for Scotland [20]. The “score” for diet quality ranges from 0% if none of the dietary goals were achieved, to 100% if all the dietary goals were achieved. The DQI is broadly based on that developed by Barton et al. [23] and modified for household purchase data [24]. KWP purchase data are recorded per household, whereas the dietary goals are set per person, and goals for some nutrients differ by age. To account for differing household composition, equalized household values were used to give per adult-equivalent values for food and drink purchases that were comparable to the dietary goals, by assuming that food and drink purchases are consumed by household members *pro rata* to estimated energy requirements. The total DQI score was calculated as the sum of the individual DQI components (Supplemental Table 1).

### Socioeconomic data

Socioeconomic information included household composition (age and gender of each household member, collected weekly), whereas socioeconomic information for the main shopper (social class, employment status), together with annual household income, was also available at less frequent intervals. For household location, KWP provides a Scottish Neighbourhood Statistics Data Zone identifier [25] for Scotland and a postcode within the Broadcasting Audience Research Board areas for the north of England consisting of border England, North East, North West, and Yorkshire [26]. The Scottish Data Zone identifier is then used to classify households in Scotland into quintiles by level of neighborhood deprivation using the Scottish Index of Multiple Deprivation (SIMD) 2020 [27] (where first quintile is the most deprived). Comparable data on deprivation were not available in KWP data for England.

### Sample size

We used data from the week ending the 30 April, 2017 to the week ending the 12 May, 2019, equivalent to 53 wk before and 54 wk after the MUP implementation. As KWP members report their purchases for periods ranging from a few months to many years, only households with ≥1 observation week in both the pre-MUP and post-MUP periods are included in the analysis. The sample is restricted to households with full socioeconomic data and with ≥1 observation week in both the pre-MUP and post-MUP periods. Inadequate data to enable weighting (n = 1759 households) or being observed in only 1 treatment period (n = 1617 households) resulted in the exclusion of 3376 households. No data imputation for missing data was conducted.

The KWP data used for these analyses include 1987 participating households in Scotland and 6064 households in the north of England (Supplemental Figure 1). Although there is no consensus on statistical inference for difference-in-differences (D-I-D) analysis [28], a conservative analysis prior to the study indicated the available sample size within the KWP data was sufficient to achieve ≥90% power to detect a 1% reduction in food spending.

### Statistical analysis

All statistical analysis was undertaken using STATA 14.2 (STATA Corp). We employed D-I-D analysis to compare nutritional components of foods and quality of diet purchased by households in Scotland (treatment group) with those purchases made by households in the adjacent north of England (control group) before and after the implementation of MUP in Scotland. This D-I-D design sets up a treatment-control comparison where the changes in north of England purchases are assumed to be a counterfactual for the changes in Scotland had the MUP policy not been implemented in Scotland. This is a well-established method for analyzing policy interventions across different jurisdictions and time periods where randomization is not possible [28]. The quasi-experimental study design supports causal inference, such that any changes observed are possible to attribute to the policy.

Many of the outcome variables have a positively skewed distribution with many zeros concentrated in the left tail of the distribution; therefore, the Poisson pseudomaximum likelihood regression model with household fixed-effects was used to perform the D-I-D analysis because it makes minimal assumptions about the distribution of the data. Time-invariant fixed effects included employment status, household income, social class, and an urban/rural dummy. Estimation was performed with Stata 14.2 using the user-written command, *ppmlhdfe* for estimating Poisson pseudomaximum likelihood with high-dimensional fixed effects [29], which allows multiple sources of heterogeneity to be controlled for. All analysis is adjusted for social economic factors including age, total number of people in the household, whether having children, spending on nonfood categories, sample observation time from baseline, and month dummies. The treatment effect was estimated from the coefficient of an exposure dummy variable (post-MUP in Scotland). To compute the effect size as a percentage change, the coefficient was transformed as exp^coefficient-1^.

As the sample of households from the north of England was substantially larger than the Scottish sample, with potential heterogeneities in observable characteristics between the 2 samples prior to the implementation of MUP, we applied entropy balancing to reweight and balance inequalities in the means and variances of the observable characteristics. This is a data preprocessing method to achieve covariate balance in observational studies by reweighting the control group [30]. These balance improvements can reduce model dependence for the subsequent estimation of treatment effects [31]. The weight was generated so that it minimized the entropy distance metric of selected covariates subject to a set of balance constraints that equated the moments of the covariate distribution (mean and variance) between the treatment and the reweighted control group. The Stata user-written package *ebalance* [31] was used to compute unit weights for reweighting selected covariates before MUP in the control groups.

We first examined the average effect of MUP on nutrient intakes and diet quality. As MUP may not have equal effects on different populations, we then examined heterogeneous effects of the MUP on households living in neighborhoods with different levels of deprivation, measured by SIMD, and on those with different levels of purchase of alcohol (≤14 units compared with over 14 units/adult/wk).

An interaction term is added between the treatment effects dummy and living in the top 2 quintiles of the SIMD (759 households in Scotland in the least deprived areas and 1228 households in more deprived areas). Note that comparable data on deprivation are not available for England. Another interaction term is between treatment effects dummy and purchasing a higher level of alcohol purchase (over 14 units/adult/wk) (n = 951, with 239 households in Scotland and 722 households in north of England).

## Results

### Sample balance at baseline

Table 1 compares the baseline characteristics (before MUP) between Scotland and north of England (unweighted and weighted). After entropy weighting, there were no significant differences in baseline characteristics between Scotland and weighted north of England. The average age of household shoppers was 50 y old, with over 70% females. Around 40% of household shoppers worked over 30 h/wk, with income mainly concentrated between £10,000 to £29,999.

**TABLE 1:** Household characteristics at first observation within the sample

Variable	Scotland - unweighted (*n* = 1987)	North of England - unweighted (*n* = 6064)	North of England -weighted (weighted *n* = 1987)
Log years in panel	1.31 (1.77) [–0.98]	1.58 (1.26) [–1.30]	1.31 (1.77) [–0.98]
Main shopper: age (y)	50.52 (194.1) [0.20]	50.10 (208.5) [0.21]	50.52 (194.1) [0.20]
Main shopper: age (y) squared/100	27.47 (214.3) [0.72]	27.18 (227.1) [0.69]	27.47 (214.3) [0.72]
Household size (number of people)	2.54 (1.60) [0.81]	2.73 (1.75) [0.77]	2.54 (1.60) [0.81]
Children (% households with ≥1 child)	0.30	0.34	0.30
Main shopper: male	0.29	0.26	0.29
Employment			
Over 30 h/wk	0.43	0.40	0.43
8–29 h/wk	0.17	0.19	0.17
Under 8 h/wk	0.02	0.02	0.02
Not working	0.12	0.12	0.12
Unemployed	0.02	0.02	0.02
Full-time education	0.01	0.00	0.01
Retired	0.23	0.25	0.23
Household income			
£0–£9999 per annum (pa)	0.07	0.18	0.07
£10,000–£19,999 pa	0.22	0.07	0.22
£20,000–£29,999 pa	0.22	0.22	0.22
£30,000–£39,999 pa	0.17	0.22	0.17
£40,000–£49,999 pa	0.12	0.12	0.12
£50,000–£59,999 pa	0.08	0.08	0.08
£60,000–£69,999 pa	0.05	0.04	0.05
£70,000+ pa	0.06	0.06	0.06
Social class			
AB	0.21	0.21	0.21
C1	0.38	0.39	0.38
C2	0.17	0.18	0.17
D	0.15	0.13	0.15
E	0.09	0.09	0.09
Household location: rural	0.21	0.14	0.21

Values in columns 2–4 indicate sample proportion unless otherwise stated

Notes: The sample size comprises 1987 households in Scotland and 6064 households in the north of England. Variance in parentheses, and skewness in brackets for continuous variables only. Social class AB: higher and intermediate managerial, administrative, and professional occupations. Social class C1: supervisory, clerical, and junior managerial, administrative, and professional occupations. Social class C2: skilled manual occupations. Social class D: semiskilled and unskilled manual occupations. Social class E: unemployed and the lowest grade occupations, often those on state benefits.

### Comparison of nutritional components and quality of diet

Table 2 summarizes the average nutritional components of food and DQI in Scotland and north of England, before and after MUP. These values suggest that the households in Scotland and north of England were similar in terms of intake of nutritional components, both before and after MUP. In terms of differences, considering the pre-MUP values, the largest proportional difference related to sugar, where sugar purchase from all sources, and sugar purchase excluding alcohol, was 5 g and 5.1 g higher per adult equivalent per day in Scotland pre-MUP.

**TABLE 2:** Mean (SD) daily energy, nutrients and foods available for consumption per adult equivalent per day (or per week for oil-rich fish), in Scotland and north of England

Outcome variables	Before MUP		Difference	After MUP		Difference
	Scotland	North of England		Scotland	North of England	
Energy (kcal)	2962 (2090)	2947 (2101)	15	2966 (2130)	2992 (2155)	–26
Energy density (kcal/100 g)	638 (415)	590 (406)	48	648 (420)	602 (401)	46
Fruit and vegetables (g)	405 (387)	400 (367)	5	402 (386)	404 (371)	–2
Fish (oil-rich fish, g/wk)	37 (136.5)	42 (144.5)	–5	37 (132)	44 (147)	–7
Meat (red and processed meat, g)	175 (230)	175 (236)	0	176 (239)	178 (241)	–2
Carbohydrate (g)	299 (215)	298 (215)	1	297 (216)	299 (216)	–2
Sugar (g)	79 (84)	74 (79)	5	76 (80)	73 (78)	3
Sugar excluding alcohol (g)	78 (83)	73 (78)	5	75 (80)	71 (77)	4
Sugar including alcohol only (g)	1 (5)	1 (5)	0	1 (4)	1 (5)	0
Fat (g)	132 (110)	130 (109)	2	134 (113)	133 (113)	1
Saturated fat (g)	52 (44)	51 (44)	1	52 (46)	51 (45)	1
Salt (g)	10 (9)	10 (9)	0	10 (9)	10 (9)	0
Fiber (g)	22 (16)	22 (17)	0	22 (17)	23 (17)	–1
DQI (%)	37 (16)	38 (16)	–1	37 (16)	38 (16)	–1

Note: The sample size comprises 1987 households in Scotland and 6064 households in the north of England.

Abbreviations: DQI, Diet Quality Index; MUP, minimum unit pricing for alcohol.

Supplemental Figure 2 also illustrates the trends of nutritional components of food and DQI during the pre-MUP and post-MUP periods for Scotland and north of England. Overall, trends of all outcomes are similar between Scotland and north of England, supporting the parallel trends assumption.

### Mean change in nutrient intakes and DQI

**FIGURE 1:**
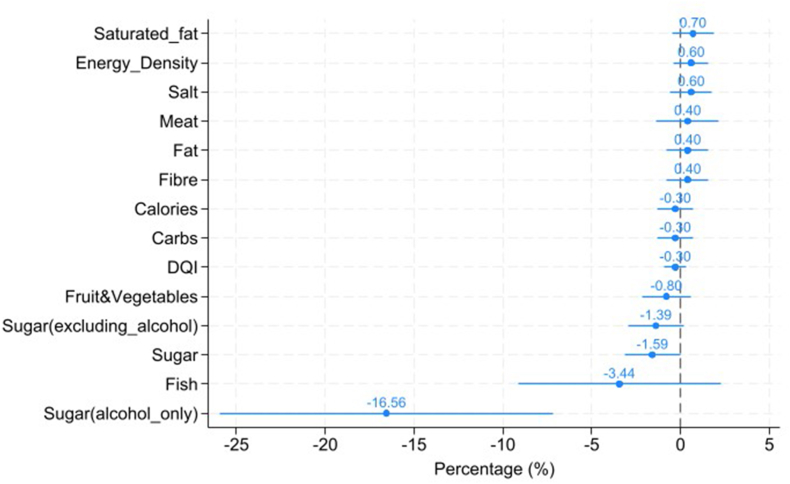
Estimated associations among MUP, nutrient intakes, and DQI score (percentage change with 95% confidence interval). The X-axis represents percentage changes based on a difference-in-differences approach. The analysis draws on data from the United Kingdom Kantar Worldpanel, covering the period from 30 April, 2017 to the week ending 12 May, 2019. The sample comprises 1987 households in Scotland and 6064 households in north of England. The estimates are derived from a weighted sample using an entropy balancing approach. The Y-axis indicates saturated fat, salt, energy density, fiber, fat, meat, dietary quality index, carbohydrate, energy, fruit and vegetables, sugar (excluding sugar from alcoholic drinks), sugar (total), fish, and sugar (from alcoholic drinks only). DQI, Diet Quality Index; MUP, minimum unit pricing for alcohol.

Figure 1 illustrates the percentage change in nutrient intakes and DQI. Table 3 extends Figure 1 by showing the exact coefficient estimates, SEs, calculated percentage changes, and level changes as calculated from the mean values and percentage change estimates. Compared with the counterfactual change in the north of England, MUP had no significant association with most nutrient intakes and DQI. However, MUP had a significant association with sugar purchase. Given that sugar is found in some alcoholic drinks, which could be affected directly by MUP, sugar intakes were classified into “sugar from all sources,” “sugar from all sources excluding alcoholic drinks,” and “sugar from alcoholic drinks only.” MUP was significantly associated with reduced total sugar purchase by 1.6% per week in Scotland [95% confidence interval (CI): –0.02%, –3.16%; *P* < 0.05], or ∼8 g per adult equivalent per week. MUP was also significantly associated with reduced purchase of sugar from alcoholic drinks by 16.6% per week (95% CI: –7.15%, –25.9%; *P* < 0.01), or ∼1.4 g less per adult equivalent per week. In addition, MUP was associated with a reduction of sugar from all sources excluding alcoholic drinks by 1.4% per week (*P* < 0.1) or ∼7 g less per adult equivalent per wk.

**TABLE 3:** Estimated associations among MUP, nutrient intakes, and DQI score

	Energy (kcal)	Energy density (kcal/100 g)	Fruit and vegetables (g)	Oil-rich fish (g/wk)	Red and processed meat (g)
Mean	2968	608	402	41.2	176
Coefficients from PPML	–0.003	0.006	–0.008	–0.035	0.004
SE	(0.005)	(0.036)	(0.007)	(0.029)	(0.009)
Percentage change (%)	–0.3	0.6	–0.8	–3.4	0.4
Level change	–8.9	3.7	–3.2	–1.4	0.7
	Carbohydrate (g)	Sugar (g)	Sugar excluding alcohol (g)	Sugar including alcohol only (g)	Fat (g)
Mean	298	74.3	73.1	1.25	131.8
Coefficients from PPML	–0.003	–0.016	–0.014	–0.181	0.004
SE	(0.005)	(0.008)	(0.008)	(0.048)	(0.006)
Percentage change (%)	–0.3	–1.6	–1.4	–16.6	0.4
Level change	–0.9	–1.2	–1.0	–0.2	0.5
	Saturated fat (g)	Salt (g)	Fiber (g)	DQI (%)	
Mean	51.3	9.91	22.4	37.9	
Coefficients from PPML	0.007	0.006	0.004	–0.003	
SE	(0.006)	(0.006)	(0.006)	(0.003)	
Percentage change (%)	0.7	0.6	0.4	–0.3	
Level change	0.4	0.1	0.1	–0.1	

Values are mean daily amounts per adult equivalent.

The sample size comprises 1987 households in Scotland and 6064 households in the north of England.

Percentage change was calculated using coefficients from PPML: exp(coefficient)−1; level change was calculated based on the mean of outcomes and percentage change: mean × [exp(coefficient)−1]. For example, level change for energy is calculated as 2968∗[exp(–0.003)]−1 = –8.9 kcal/person/d.

All analyses are adjusted for social economic factors, including age, total people in household, whether having children, spending on nonfood categories, and month dummies.

*P* values were calculated using *t*-tests and are reported in the text where *P* < 0.1.

Abbreviations: DQI, Diet Quality Index; MUP, minimum unit pricing for alcohol; PPML, Poisson pseudomaximum likelihood.

### Heterogeneous association between MUP, nutrient intakes and DQI

Supplemental Table 2 shows the association between MUP and outcomes by households in the bottom 3 quintiles of SIMD and those in the top 2 quintiles of SIMD. Overall, MUP had no significant associations among the 2 groups across most of the measures. However, households from the bottom 3 quintiles of SIMD reduced sugar intake from alcohol more than those from the top 2 quintiles of SIMD (*P* < 0.01). Also, households from the bottom 3 quintiles of SIMD reduced purchase of oil-rich fish significantly more than those from top 2 quintiles of SIMD (*P* < 0.05).

Supplemental Table 3 compares the association between MUP and outcomes by households with a high level of alcohol purchase (>14 units/adult/wk) and moderate level of alcohol purchase (1-14 units/adult/wk). Again, among most measures, MUP had no significant associations among the 2 groups. Notably, households with a higher level of alcohol purchase were associated with reduced sugar intake from alcohol significantly more than those with a moderate level of alcohol purchase (*P* < 0.01).

## Discussion

Our findings suggest that, compared with the counterfactual north of England, the implementation of MUP for alcohol was not significantly associated with adverse nutritional outcomes in Scotland apart from a small increase in energy density (*P* < 0.10). There were no significant associations with changes in overall diet quality or nutrients except for sugar. The significant association between MUP and reduced sugar intake is a potentially beneficial outcome, observed both for sugar from all sources and sugar from alcohol only. Moreover, in subgroup analysis, a reduction in sugar is observed for households in the more deprived areas.

The estimated effect size of the policy on sugar purchased may appear modest at ∼8 g/adult equivalent per week on average, especially given the finding that there is no improvement in the diet quality score for sugar intakes. Yet this estimated reduction is more than comparable to the impact of policies specifically targeting sugar consumption in the United Kingdom, such as the Soft Drinks Levy introduced prior to MUP. An evaluation of the levy, also using Kantar data, estimated a reduction in sugar purchased from soft drinks of 8.0 g/household/wk [32]. Previous voluntary action by Public Health England and the food industry to reduce sugar content had not reduced sugar purchased per person [33].

The association between MUP and purchase of added sugar from alcoholic drinks is negative and statistically significant, both across the entire population and within particular subgroups, notably those purchasing higher levels of alcohol and those living in more deprived areas. These findings not only confirm the drop in alcohol purchases in Scotland post-MUP reported in previous studies [[34], [35], [36]] but also support the heterogeneous impacts found in those studies [34,35], where the high purchase households decreased their alcohol purchasing significantly more. Furthermore, a report by Public Health Scotland [36] showed that the biggest reduction in consumption following MUP was of cider and perry, which are drinks with high sugar content.

The potential health impacts of reduced sugar consumption appear considerable [37]. Using data from the National Diet and Nutrition Survey, the authors estimated that a 20% reduction in sugar intake from baseline levels would be projected to produce National Health Service cost savings of £124 million for males and £162 million for females, and increased health benefits through additional Quality-Adjusted Life Years gained of between 23,874 (males) and 27,855 (females), measured over a 10-y period. The effects were generated through a reduction of 19 kcal/d for adults and a weight reduction of between 1.5 and 1.8 kg. The health benefits accrued would be generated from avoided cases of cardiovascular disease, stroke, diabetes, cirrhosis, and cancer, with the largest cases avoided being generated from diabetes. However, the modeling does not take into account consumer response in the form of additional consumption of other products, or indeed additional consumption of those products that were reformulated. In addition, the estimates rely on self-reported cross-sectional associations between risk factors and disease. Therefore, it is possible that the estimates are upper limits of the potential impacts.

Regarding oil-rich fish purchases, our finding of reductions in purchases among the subgroup of people from more deprived households is consistent with a previous study that used the same KWP data and found a significant reduction across the population in the volume of fish purchases following MUP [18]. It is difficult to pinpoint reasons why MUP would be associated with such a change, which suggests this finding is either artefactual or driven by unobservable factors. In addition, the data for fish are highly skewed, and the consumption of oily fish by the most deprived groups was reported to be half the level of less deprived groups prior to MUP being introduced [38].

A strength of this study is the use of a robust D-I-D design and a large dataset with detailed dietary information. The parallel trends assumption holds in our sample prior to the introduction of MUP (shown in Supplemental Figure 2). Although the Soft Drinks Industry Levy (sugar tax) was implemented in the whole United Kingdom less than a month before MUP came into effect, the sugar tax affected both Scotland and the north of England equally.

There are some limitations of our study due to the nature of the KWP data. First, we have no information on purchases consumed outside the home; however, we are not aware of any changes other than MUP that would have a differential impact on this in Scotland compared with the north of England. Second, we implicitly assume that households consume all food and drinks bought in that week. This is unlikely because some food and drinks might be bought and kept longer for future consumption, and some could end up as food waste, particularly among fresh products. Indeed, a systematic review [39] indicates that people often follow routines of buying more food than needed and overprovisioning of food is 1 of the main reasons for food waste in private households. Although we are aware that wasted food causes sizable nutrient loss [40], purchasing data such as KWP do not provide any information on food waste in the households. However, with weekly panel data collection, the issues of time inconsistency between purchase and consumption should be mitigated because households in Scotland and the north of England should have similar and stable patterns in food and drink consumption (in terms of days between purchasing and consuming) after their purchases.

In terms of the generalizability of the results, although the sample provided by the KWP is designed to be representative of the population, it does not fully cover the heaviest purchasers of alcohol. In a qualitative study of people who are drinking at harmful levels [over 35 units for females (50 units for men) per week] or dependent on alcohol, some have reported reducing expenditure on food and, in some cases, using food banks to access free food [41]. Equally, some of those in the study reported being more likely to seek treatment for their drinking. Furthermore, the data only cover purchases of food and alcoholic drinks brought into the home, and excludes food takeaway purchases and restaurant meals, which may limit the generalizability of results to those households that have only limited and occasional out-of-home purchasing.

One remaining concern is the effect of MUP on the shares of off-trade sales and on-trade sales (e.g., pubs and clubs) of alcohol in Scotland. Although the MUP applied to all types of alcohol sales in Scotland, it could narrow the price difference between on-trade sales and off-trade sales because the price of on-trade sales tended to be higher before the MUP introduction in May 2018. A reducing gap in Scotland could encourage a substitution from off-trade purchases toward on-trade consumption, and hence could partially reverse the negative impact of MUP on sugar intakes, especially sugar from alcoholic drinks. However, aggregated level data published by Public Health Scotland [36] depicts similar trends in on-trade sales of alcohol (liters of pure alcohol per adult) between Scotland and England during 2018 and 2019, decreasing slightly from the 2017 level. Thus, our estimated effects should not be seriously altered and can be considered a conservative upper bound of the reduction of sugar intakes arising from the policy.

In conclusion, the analysis presented here suggests that the implementation of MUP made little difference to nutrition from food purchased to eat at home, except for a possible reduction in sugar consumption. The potential for further impact should, however, continue to be considered as part of any future review of changes to MUP policy.

## Author contributions

The authors’ responsibilities were as follows – A Ludbrook, PM, SW: overall study design; NG, DK, A Leckcivilize, SW: data preparation; DK, NG, A Leckcivilize: statistical analysis; NG, A Ludbrook, A Leckcivilize, PM, SW: writing and/or editing manuscript; and all authors: commented on previous versions of the manuscript, read and approved the final manuscript.

## Data availability

Data described in the manuscript, code book, and analytic code will not be made available because the funding agreement precludes this.

## Ethics Statement

This project used anonymized secondary data for which informed consent was received by the original data collectors. No further ethical approval was required for this project.

## Funding

The study was funded by a research grant from the Chief Scientist Office (CSO) of the Scottish Government Health and Social Care Directorates, HIPS Grant Number HIPS/19/01, following a competitive peer review process. The views expressed in the article are those of the authors only and not those of the funder.

## Conflict of interest

The authors declare no conflict of interest.
