# Supplementary material for: Reduction in sugar intake after the introduction of minimum unit pricing for alcohol in Scotland: a difference-in-differences analysis
Source: Am J Clin Nutr. 2025 Nov 27;123(2):101128. doi: 10.1016/j.ajcnut.2025.101128 (PMC12917215; doi:10.1016/j.ajcnut.2025.101128)
Supplement: multimedia component 1 [file mmc1.docx]

**Leckcivilize et al.**

**Reduction in Sugar Intake after the Introduction of Minimum Unit Pricing for Alcohol in Scotland: a Difference-in-Differences Analysis**

**Online Supplementary**

**Supplemental Table 1: Components of the Diet Quality Index and scoring criteria**


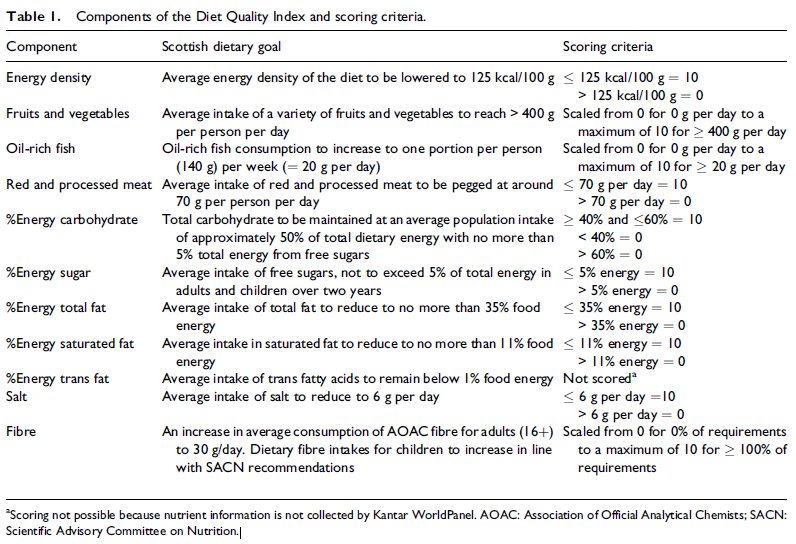


**Note:** The total estimated energy requirement for each household was calculated by summing the requirements for adults and children, accounting for age and sex (42). The estimated energy requirement of children under 2 years old was not included. A reference energy requirement for all adults was estimated as the average for 19–59 years old males and females (2223 kcal per day) (42). This allowed food and nutrient intake to be estimated per adult equivalent from combined household purchases. For example, if a household of two elderly adults, with an estimated energy requirement of 4254kcal per day, purchased 300g of fruit in a week this would be 300 / 7 / (4254 / 2223) = 22.4g per adult equivalent per week.

**Supplemental Table 2: Heterogeneous associations between MUP, nutrient intake and DQI score (percentage change) by deprivation level in the neighbourhood area**

|  | Energy | | Energy density | | | Fruit & Vegetables | | Oil-rich fish | | Red and processed meat | |
| --- | --- | --- | --- | --- | --- | --- | --- | --- | --- | --- | --- |
| Post-MUP | 0.010 | | -0.003 | | | 0.011 | | 0.008 | | 0.012 | |
|  | (0.006) | | (0.004) | | | (0.008) | | (0.032) | | (0.013) | |
| Scotland Post-MUP | -0.003 | | 0.006 | | | -0.009 | | -0.082 | | 0.012 | |
|  | (0.006) | | (0.004) | | | (0.009) | | (0.041) | | (0.011) | |
| Scotland Post-MUP*Top two SIMD quintiles | 0.001 | | 0.001 | | | 0.003 | | 0.107 | | -0.023 | |
|  | (0.009) | | (0.006) | | | (0.012) | | (0.050) | | (0.016) | |
|  | |  | |  | |  | | |  | |  |
|  | |  | |  | |  | | |  | |  |
|  | | Carbohydrate | | Sugar | | Sugar excluding alcohol | | | Sugar including alcohol only | | Fat |
| Post-MUP | | -0.000 | | -0.004 | | -0.006 | | | 0.119 | | 0.015 |
|  | | (0.006) | | (0.009) | | (0.009) | | | (0.039) | | (0.007) |
| Scotland Post-MUP | | -0.004 | | -0.021 | | -0.018 | | | -0.230 | | 0.004 |
|  | | (0.006) | | (0.009) | | (0.009) | | | (0.068) | | (0.007) |
| Scotland Post-MUP*Top two SIMD quintiles | | 0.003 | | 0.013 | | 0.011 | | | 0.140 | | 0.001 |
|  | | (0.009) | | (0.014) | | (0.015) | | | (0.073) | | (0.010) |
|  | |  | |  | |  | | |  | |  |
|  | |  | |  | |  | | |  | |  |
|  | | Saturated fat | | | Salt | | Fibre | | DQI | |  |
| Post-MUP | | 0.010 | | | 0.000 | | 0.011 | | 0.003 | |  |
|  | | (0.008) | | | (0.008) | | (0.007) | | (0.004) | |  |
| Scotland Post-MUP | | 0.009 | | | 0.009 | | 0.003 | | -0.004 | |  |
|  | | (0.007) | | | (0.008) | | (0.007) | | (0.004) | |  |
| Scotland Post-MUP*Top two SIMD quintiles | | -0.005 | | | -0.007 | | 0.002 | | 0.003 | |  |
|  | | (0.010) | | | (0.011) | | (0.009) | | (0.005) | |  |

The analysis draws on data from the UK Kantar Worldpanel, covering the period from April 30, 2017 to the week ending May 12, 2019. The sample comprises 1,987 households in Scotland and 6,064 households in Northern England. The estimates are derived from a weighted sample using entropy balancing approach. Category-level models include age of shopper, age of shopper squared/100, total people in household, children dummy, log years in panel, non-food spend in week, and month of purchase. Robust standard errors in parentheses.

MUP=Minimum Unit Pricing for alcohol.

DQI=Diet Quality Index.

SIMD=Scottish Index of Multiple Deprivation.

**Supplemental Table 3: Heterogeneous associations between MUP, nutrient intake and DQI score (percentage change) by level of alcohol purchase**

|  | Energy | Energy density | | Fruit & Vegetables | Oil-rich-fish | | Red and processed meat |
| --- | --- | --- | --- | --- | --- | --- | --- |
| Post-MUP | 0.010 | -0.003 | | 0.011 | 0.008 | | 0.012 |
|  | (0.006) | (0.004) | | (0.008) | (0.032) | | (0.013) |
| Scotland Post-MUP | -0.002 | 0.007 | | -0.008 | -0.041 | | 0.006 |
|  | (0.005) | (0.004) | | (0.008) | (0.031) | | (0.010) |
| Scotland Post-MUP*High alcohol purchase | -0.009 | -0.002 | | 0.003 | 0.038 | | -0.015 |
|  | (0.014) | (0.008) | | (0.019) | (0.071) | | (0.021) |
|  |  |  | |  |  | |  |
|  | Carbohydrate | Sugar | | Sugar excluding alcohol | | Sugar including alcohol only | Fat |
| Post-MUP | -0.000 | -0.004 | | -0.006 | | 0.118 | 0.015 |
|  | (0.006) | (0.009) | | (0.009) | | (0.039) | (0.007) |
| Scotland Post-MUP | -0.003 | -0.018 | | -0.017 | | -0.050 | 0.004 |
|  | (0.006) | (0.008) | | (0.008) | | (0.034) | (0.006) |
| Scotland Post-MUP*High alcohol purchase | 0.002 | 0.013 | | 0.027 | | -0.251 | -0.001 |
|  | (0.015) | (0.023) | | (0.024) | | (0.085) | (0.016) |
|  |  |  | |  | |  |  |
|  | Saturated fat | | Salt | Fibre | DQI | |  |
| Post-MUP | 0.010 | | 0.000 | 0.011 | 0.003 | |  |
|  | (0.008) | | (0.008) | (0.007) | -0.004 | |  |
| Scotland Post-MUP | 0.007 | | 0.008 | 0.004 | -0.004 | |  |
|  | (0.006) | | (0.006) | (0.006) | -0.003 | |  |
| Scotland Post-MUP*High alcohol purchase | -0.005 | | -0.011 | -0.004 | 0.004 | |  |
|  | (0.016) | | (0.020) | (0.015) | -(0.009) | |  |

The analysis draws on data from the UK Kantar Worldpanel, covering the period from April 30, 2017 to the week ending May 12, 2019. The sample comprises 1,987 households in Scotland and 6,064 households in Northern England. The estimates are derived from a weighted sample using entropy balancing approach. Category-level models include age of shopper, age of shopper squared/100, total people in household, children dummy, log years in panel, non-food spend in week, and month of purchase. Robust standard errors in parentheses.

MUP=Minimum Unit Pricing for alcohol.

DQI=Diet Quality Index.

**Supplemental Figure 1. Participant Flow Chart.**

**Supplemental Figure 2. Trends of nutritional components of food and DQI between Scotland (red line) and North of England (blue line)**

|  |  |
| --- | --- |
|  |  |
|  |  |
|  |  |
|  |  |
|  |  |
|  |  |

*Yellow line is the date of MUP implementation; X-axis is weeks before and after MUP; for Y-axis, see Table 2.

MUP=Minimum Unit Pricing for alcohol.

DQI=Diet Quality Index.

Reference

[42] Scientific Advisory Committee on Nutrition, Dietary reference values for energy, The Stationery Office, 2012 May 2. https://assets.publishing.service.gov.uk/media/5a7edb37ed915d74e33f2d8f/SACN_Dietary_Reference_Values_for_Energy.pdf. Date cited: 11/12/24.
